# Paliperidone‐Induced Catatonia: Case Series and Review of Literature

**DOI:** 10.1002/ccr3.71571

**Published:** 2025-12-07

**Authors:** Sikandar Saeed, Sakshi Prasad, Arham Javaid, Elzar Matmusaeva, Marcos Rocha, Resha Karani, Samkit Jain, Khai Tran, Sasidhar Gunturu

**Affiliations:** ^1^ BronxCare Health System New York New York USA; ^2^ School of Medicine American University of the Caribbean Cupecoy Sint Maarten; ^3^ American University of Antigua College of Medicine Coolidge Antigua and Barbuda; ^4^ Vinnytsya National Medical University Vinnitsiya Ukraine

**Keywords:** case report, catatonia, long acting injectable, paliperidone, psychopharmacology

## Abstract

Paliperidone, a second‐generation antipsychotic commonly used to treat schizophrenia and schizoaffective disorder, has been rarely associated with inducing catatonia, a severe neuropsychiatric syndrome characterized by motor disturbances and altered mental status. We present two cases demonstrating variable onset of paliperidone‐induced catatonia: a 20‐year‐old male with acute symptoms following intramuscular loading doses and a 24‐year‐old female with a delayed presentation. Both cases highlight the clinical challenge of timely recognition, which is critical to prevent serious complications such as dehydration and respiratory failure. Assessment using the Bush‐Francis Catatonia Rating Scale (BFCRS) guided treatment with benzodiazepines and antipsychotic adjustments, resulting in significant symptom improvement. This case series underscores the need for heightened awareness of this rare adverse effect to facilitate early diagnosis and optimize outcomes in patients receiving paliperidone.

## Key Clinical Message

Paliperidone, a potent second‐generation antipsychotic, can rarely induce catatonia characterized by motor and behavioral disturbances. Early recognition and treatment with benzodiazepines, alongside antipsychotic adjustment, are critical to prevent severe complications and improve patient outcomes.

## 1 Introduction

Paliperidone (Invega) is a second‐generation antipsychotic (SGA) primarily prescribed for the treatment of schizophrenia and schizoaffective disorder. Approved by the US Food and Drug Administration (FDA) in 2006, it is the major active metabolite of risperidone, and works by blocking both dopamine D2 and serotonin 5‐HT2A receptors, which contribute to its therapeutic effects [1]. Paliperidone exhibits a higher affinity for dopamine receptors than risperidone, which may lead to improved control of schizophrenia's positive symptoms. Its longer half‐life also reduces the need for frequent dosing, helping to improve patient adherence to treatment. Unlike risperidone, paliperidone undergoes minimal hepatic metabolism, making it a preferable option for individuals with liver impairment [2].

Although generally well tolerated, paliperidone has been associated with a range of adverse effects, including extrapyramidal symptoms, hyperprolactinemia, cardiovascular complications, and, in rare cases, neuroleptic malignant syndrome [2]. A growing concern is its potential to induce catatonia, a severe neuropsychiatric syndrome characterized by motor disturbances such as stupor, mutism, posturing, rigidity, and agitation. According to the DSM‐5, catatonia is diagnosed when at least three of the following symptoms are present: stupor, mutism, posturing, catalepsy, or echolalia [3]. Catatonia presents in different forms, including retarded catatonia (marked by withdrawal and immobility) and excited catatonia (characterized by excessive and purposeless motor activity). Malignant catatonia, a life‐threatening subtype, involves autonomic instability, hyperthermia, and severe rigidity [4]. Timely recognition of antipsychotic‐induced catatonia is crucial, as untreated cases can result in severe complications such as dehydration, respiratory failure, rhabdomyolysis, and death.

This paper presents a case series of two patients who experienced moderate to severe catatonia after receiving an intramuscular loading dose of Invega Sustenna. The patients had BFCRS scores of 15 and 11, respectively. By examining the pathophysiological mechanisms, clinical features, and management approaches for paliperidone‐induced catatonia, the goal of this study is to enhance early detection, prevention, and treatment of this rare yet significant condition, ultimately improving patient outcomes.

## 2 Case History/Examination

### 2.1 Case 1

A 20‐year‐old male originally from Jamaica with a psychiatric history of schizophrenia was brought to the emergency room for erratic behavior, verbal and motor hypoactivity, sialorrhea, generalized body stiffness, and slowed speech. The patient's mother noted that he had been acting like this since being discharged from another psychiatric hospital's inpatient unit 1 day ago, where he was admitted due to aggressive behavior at home. He was admitted to that hospital's inpatient psychiatry unit for a period of 3 weeks and received Invega Sustenna (Paliperidone) loading doses on the day of discharge. Due to concerns for altered mental status, the patient was transferred to the medical ED.

While in the ED, the patient was treated with IM Benztropine 1 mg, and IM Lorazepam 2 mg, with partial resolution of symptoms. The patient was admitted to the medical inpatient unit and assessed by the Consultation‐Liaison psychiatry team. On evaluation, he appeared catatonic with stereotyped posturing of arms at a ninety‐degree angle to his body, with psychomotor/speech slowing, oddly related, and repetitive, slurred speech. He exhibited a disorganized thought process during the initial interview and was unable to state why he came to the hospital. His Bush‐Francis score for catatonia was 15 at this time. He was transferred to inpatient psychiatry following this assessment. During his 2‐day stay in the inpatient medical unit, his catatonia mildly improved, with a BFCRS of 12.

On Day 3, while in the inpatient psychiatry unit, he still presented with stiffness, stereotyped posturing of the upper limbs, slowed speech, disorganized thought process with grandiose thought content, and reported auditory hallucinations. His mother was contacted for collateral, and stated that the current presentation was atypical for the patient, whom she reported is usually fairly active and communicative, and noted that he received the Invega series with 234 mg and 156 mg for psychotic symptoms during his recent prior psychiatric admission, the last dose being the day before his current admission with catatonic symptoms. She stated that she was concerned because the patient was already exhibiting some symptoms of catatonia on discharge, which worsened after he got home.

#### 2.1.1 Treatment

The patient was started on oral Risperidone 1 mg twice daily along with Benztropine 1 mg twice daily and Valproate 500 mg twice daily. He was also given oral Lorazepam 1 mg twice daily.

#### 2.1.2 Outcome & Follow Up

While on this regimen, the patient significantly improved in terms of catatonic symptoms, returning to normal ambulation, with no stiffness, and reverting to a regular rate of speech. The Lorazepam was tapered off starting after 4 days of 1 mg twice daily dosing, and completely tapered off after 10 days of treatment. The patient also reported abatement of his auditory hallucinations, with some residual grandiosity. His mother stated that she felt he was at his baseline, and he was discharged with oral medications and outpatient follow‐up.

### 2.2 Case 2

A 24‐year‐old African American female with a psychiatric history of schizophrenia was admitted to the psychiatric hospital after being found at a train station with disorganized behavior and auditory hallucinations endorsing voices that were “torturing” her. Upon admission, she was noted to have poor oral intake and difficulty swallowing, which prompted a transfer to the medical unit for stabilization.

On psychiatric assessment, the patient appeared stiff, slow‐moving, and unsteady, demonstrating significant motor symptoms consistent with catatonia. She was able only to follow basic instructions, and had difficulty eating and drinking, spilling liquids from her mouth. Bush‐Francis score was 11. Lorazepam 1 mg twice a day was continued and her condition was closely monitored. After improved oral intake, the patient was transferred back to inpatient psychiatry.

Per patient's last discharge summary from an outside hospital, she was administered Invega Sustenna 234 mg intramuscularly, followed by a second loading dose of 156 mg, 6 weeks prior to this admission. She did not have catatonic symptoms prior to the LAI; catatonic symptoms were not mentioned in the discharge summary, nor reported by the patient. Oral paliperidone was discontinued, and risperidone was started. Abdominal imaging was done to rule out ovarian teratomas to screen for autoimmune encephalitis, which was negative.

#### 2.2.1 Treatment

The patient was treated with Risperidone 1 mg twice a day (BID) for psychosis, titrated to 2 mg BID, along with Lorazepam, titrated to 1 mg BID for catatonic symptoms. She was also started on Sertraline 50 mg daily, and Benztropine 1 mg BID to manage cognitive, mood, and extrapyramidal symptoms.

#### 2.2.2 Outcome & Follow Up

After 3 weeks of treatment, her motor symptoms showed significant improvement, with near‐total resolution, allowing for the discontinuation of Lorazepam with continuation of Risperidone and reduction in auditory hallucinations. Prior to discharge, she was given Risperidone Consta 25 mg IM with a follow‐up outpatient appointment scheduled. On a six‐month evaluation, the patient continues to follow up outpatient with no reported side effects.

## 3 Conclusion

This case series aims to enhance awareness of drug‐induced catatonia, particularly cases associated with paliperidone. A review of existing literature on medication‐induced catatonia reveals both consistencies and discrepancies in reported incidence, risk factors, and management strategies. Prior studies have documented antipsychotic‐induced catatonia, particularly with first‐generation agents [5], but cases linked to second‐generation antipsychotics, such as paliperidone, remain less frequently reported. Our findings align with existing reports that highlight the importance of early recognition and withdrawal of the offending agent. However, variations exist in the response to benzodiazepines and other treatment options, with some studies suggesting prolonged symptom resolution despite standard interventions.

## 4 Discussion

### 4.1 Drug‐Induced Catatonia and the Role of Paliperidone

Most psychiatric disorders may simply cause catatonia, although it does appear that medications themselves can either cause or unmask an underlying predisposition to catatonia as well [7]. Drug‐induced catatonia has mostly been reported with psychotropic drugs, including fluphenazine, haloperidol, risperidone, and clozapine, non‐psychotropic drugs such as steroids, disulfiram, ciprofloxacin, several benzodiazepines, as well as drugs of abuse, including phencyclidine, cannabis, mescaline, LSD, cocaine and ecstasy [6]. While it is well established that SGAs have a lower risk of extrapyramidal side effects (EPS) than first‐generation antipsychotics (FGAs), SGAs, including paliperidone, have been known to cause catatonic symptoms in a select few patients [8]. FGAs, especially higher potency drugs such as haloperidol have long been associated with catatonic reactions. This can be attributed to their strong antagonism against the dopamine D2 receptors [4]. Case reports and pharmacological surveillance data suggest that paliperidone‐induced catatonia is uncommon but occurs more frequently than with other SGAs, such as quetiapine or clozapine. This may be attributed to their weaker D2 antagonism [9]. Additionally, certain patient populations, including those with pre‐existing neurological or genetic predispositions in dopamine receptor sensitivity are at a higher risk of developing catatonia as a side effect. Research has found an association between the dopamine D4 receptor 48‐bp repeat polymorphism and catatonic schizophrenia. Patients carrying the DRD4 D4.2 and D4.3 alleles were more frequently diagnosed with catatonic schizophrenia compared to other schizophrenic cases and controls. This genetic predisposition could potentially increase the risk of catatonia when treated with dopamine receptor antagonists such as paliperidone [10].

### 4.2 Comparison of Case Findings With Existing Literature

Most reported cases, including those by Johnson et al. [18], Tu et al. [19], and Coffey [20], describe catatonia occurring in adult patients with schizophrenia following paliperidone administration, either via intramuscular long‐acting injections or oral dosing. Similarly, our two patients—one male and one female—developed catatonic symptoms after paliperidone loading doses, consistent with the temporal relationship seen in prior reports. Johnson et al. describe a relatively rapid onset of catatonia within 5 days of intramuscular loading doses, paralleling the acute presentation in our 20‐year‐old male patient who developed symptoms within a day after discharge. These cases have been described in Table 1.

**TABLE 1:** Published literature on catatonia induced by paliperidone.

Study	Type of study	Demographics	Dosage and mode of administration	Intervention	Outcome
Johnson et al. (2023) [18]; USA	Case Report	27 year old male, Schizophrenia	Patient received paliperidone palmitate loading doses 234 mg intramuscularly and 156 mg intramuscularly on the 1st and 5th day	Oral dose of Ativan 2 mg twice daily initiated. Risperidone 3 mg twice daily was changed to an oral dose of aripiprazole 10 mg daily	Resolution of symptoms after 4 days
Tu et al. (2016) [19]; Taiwan	Case Report	28 year old, Schizophrenia	Initiation of paliperidone 9 mg once a day	Partial Resolution with biperiden injection. Oral lorazepam was up‐titrated to 3 mg/day and paliperidone was discontinued	The patient was discharged under her baseline mental status with quetiapine 75 mg/day 3 weeks after admission.
Coffey, (2012) [20]; USA	Case Report	79 year old Female, paranoid schizophrenia	24 h after receiving her sixth monthly 156‐mg injection of paliperidone palmitate	A continuous lorazepam infusion of 16 mg per 24 h reduced her Bush Francis Catatonia Rating Scale score from 25 to 12	Partial resolution after 12 ECT treatments
McKeown et al. (2010) [6]; USA	Case Report	84 year old female, MDD and Anxiety	Catatonia was observed after Single dose paliperidone 3 mg, administered for worsening anxiety	Intravenous diphenhydramine, 25 mg and benztropine, 0.5 mg were given for dystonia without any change	Self‐resolution of symptoms 1 day later
Xiong et al. (2009) [21]; USA	Case Report	51 year old male with Schizophrenia and Lewy Body Dementia	Risperidone, 0.5 mg twice daily, was changed to paliperidone, 6 mg once daily. Patient developed Catatonia 1 day after this regime	Failed trial of lorazepam 8 mg daily. All medications discontinued	Symptoms resolution post discontinuation
Mall et al. (2008) [22]; USA	Case Report	56 year old female with Schizophrenia	Catatonic 1 day after Risperidone Consta 25 mg LAI	Medications were discontinued, and she was prescribed 1 mg lorazepam (Ativan) plus 1 mg benztropine (Cogentin) 3 times daily intramuscularly	Resolution after 7 days

However, variations in the timing of onset are noteworthy. While our male patient had an acute presentation, our female patient's symptoms manifested 6 weeks post‐injection, resembling the delayed‐onset catatonia described by Yaylaci et al. [11]. This spectrum of onset timing, from immediate to delayed, complicates early diagnosis and suggests that paliperidone‐induced catatonia may develop via multiple mechanisms or in the context of varying individual susceptibilities.

Symptom profiles also show variability. Our cases presented with motor slowing and rigidity, echoing the rigidity and posturing reported by [20], who documented severe motor symptoms including stupor and mutism in an elderly female patient. In contrast, McKeown et al. [6] reported transient, self‐resolving catatonia in a patient treated with a low dose (3 mg oral) of paliperidone, highlighting a milder clinical spectrum. Our female patient's presentation included notable features such as poor oral intake and dysphagia, which prompted an extensive workup excluding autoimmune encephalitis, emphasizing the need for thorough differential diagnosis in atypical or protracted presentations.

Management strategies across cases generally involved benzodiazepine administration, consistent with established treatment guidelines. Johnson et al. used oral lorazepam, and partial symptom relief with high‐dose lorazepam and eventual electroconvulsive therapy (ECT) was critical in Coffey's case with a severe presentation. Our cases also responded to benzodiazepine therapy and careful antipsychotic adjustment, reinforcing benzodiazepines as the cornerstone for treatment. Notably, some patients required discontinuation or switching of antipsychotics, as in Xiong et al. [21] where symptoms resolved only after cessation. It is worth pointing out that the treatment of catatonia was effective even though LAIs were implicated which were presumably still present while improvement occurred.

### 4.3 Importance of Early Recognition and Diagnostic Evaluation

Early recognition of catatonia is crucial, as its symptoms carry significant prognostic and therapeutic implications. When a patient presents to the emergency department (ED) with symptoms of catatonia, it is crucial to perform a thorough assessment and consider a broad range of potential diagnoses. These symptoms should not be immediately attributed to a primary psychiatric illness without first excluding more common and treatable medical causes. A comprehensive workup may involve laboratory and imaging studies such as a complete blood count, metabolic panel, thyroid function tests, anemia panel, urinalysis, urine drug screen, cerebrospinal fluid (CSF) analysis, antibody titers, brain imaging (CT or MRI), electroencephalogram (EEG), and other investigations guided by the patient's clinical history and physical examination findings [6]. In any patient experiencing significant decline in psychomotor function and overall responsiveness, and no medical cause found on laboratory and imaging studies, psychiatric illness, including drug‐induced catatonia should be considered as a potential cause. Individuals admitted to psychiatric units with severe mental health conditions—such as major depression, bipolar disorder, psychotic disorders, or autism spectrum disorder—should undergo routine screening for catatonia. The most reliable and specific screening tools are the Rogers Catatonia Scale, the Bush‐Francis Catatonia Rating Scale (BFCRS), the Northoff Catatonia Rating Scale, and the Braunig Catatonia Rating Scale [12].

### 4.4 Treatment Approaches for Drug Induced Catatonia

Most important approach in the treatment of drug‐induced catatonia is a proper medication reconciliation of prescribed medications to evaluate for their potential to induce catatonic symptoms and to discontinue if possible [12]. Several case reports have documented that discontinuation of paliperidone in patients diagnosed with paliperidone‐induced catatonia led to symptom improvement without the need for additional interventions [6]. If catatonia persists despite discontinuing the triggering agent and providing supportive care, benzodiazepines are the preferred initial pharmacologic treatment and may be helpful as a diagnostic probe as well. A positive Lorazepam Challenge Test supports the diagnosis of catatonia. It is believed that benzodiazepines are positive allosteric modulators of GABA‐A receptors and will correct deficient GABA‐ergic function in the orbitofrontal cortex contributing to a quick positive response and improvement of catatonia symptoms in patients [12]. Lorazepam is typically started at 2–6 mg/day and may be increased to 12–16 mg/day. A response is usually seen within 3–7 days but may be gradual. The optimal duration of benzodiazepine treatment for catatonia remains uncertain, though they are usually tapered once the underlying condition resolves. However, tapering may trigger symptom recurrence, requiring prolonged use. Major other literature confirms Lorazepam and electroconvulsive therapy (ECT) as the primary treatment options for catatonia, but approximately 30% of patients may not respond to these therapies [13]. ECT is considered a first‐line intervention for neuroleptic malignant syndrome, malignant catatonia, and delirious catatonia. Its therapeutic effects are attributed to increased cerebral blood flow to the orbitofrontal and parietal cortices, enhancing GABA activity and receptor expression. In cases where benzodiazepines are ineffective, ECT serves as a definitive treatment, with reported response rates ranging from 80% to 100%. The number of ECT sessions can vary based on the patient's individual response rates and cannot be predicted exactly. Studies show patients may require several ECT treatments with at least six ECT sessions in order to see symptom relief [14].

### 4.5 Alternative and Adjunctive Therapies for Drug Induced Catatonia

Typical antipsychotics like haloperidol are generally avoided due to the risk of worsening symptoms. They entail risk of NMS, but are more likely to be of some value along with benzodiazepines in catatonia associated with schizophrenia. Atypical antipsychotics—such as risperidone or olanzapine, clozapine—may be cautiously considered in patients who don't respond to initial treatments and used off‐label to manage treatment‐resistant catatonia. They should be used in combination with a benzodiazepine, and a low potency agent should be used, although their mechanism of action is not clearly understood [15, 16]. Among other drugs used in the treatment of catatonia, including paliperidone‐induced catatonia, NMDA negative allosteric modulators (amantadine and memantine) and anticonvulsants—sodium channel blockers, which selectively block high‐frequency discharges (carbamazepine, valproate and topiramate) have also shown some benefit, though their onset tends to be slower compared to benzodiazepines ([6, 15]).

Repetitive Transcranial Magnetic Stimulation (rTMS) is gaining recognition as a potential treatment option for catatonia, particularly in cases resistant to standard therapies. Like ECT, it delivers targeted brain stimulation but has the advantage of not requiring anesthesia or causing cognitive side effects [17].

## Author Contributions

**Sikandar Saeed:** conceptualization, data curation, methodology, writing – original draft. **Arham Javaid:** conceptualization, data curation, validation, visualization, writing – original draft. **Elzar Matmusaeva:** formal analysis, funding acquisition, methodology, software, writing – original draft, writing – review and editing. **Marcos Rocha:** funding acquisition, investigation, methodology, supervision, writing – original draft, writing – review and editing. **Resha Karani:** data curation, formal analysis, funding acquisition, visualization, writing – original draft, writing – review and editing. **Samkit Jain:** methodology, writing – review and editing. **Sakshi Prasad:** conceptualization, data curation, formal analysis, supervision, validation, writing – original draft, writing – review and editing. **Khai Tran:** investigation, methodology, writing – original draft, writing – review and editing. **Sasidhar Gunturu:** data curation, formal analysis, funding acquisition, visualization, writing – original draft, writing – review and editing.

## Funding

The authors have nothing to report.

## Ethics Statement

The study was performed in accordance with the ethical standards of the Declaration of Helsinki and its later amendments, 1964. Ethical approval was obtained by the ethics committee of BronxCare Health System, New York, NY, USA.

## Consent

Written informed consent was obtained from both the patients to publish this case report in accordance with the journal's patient consent policy.

## Conflicts of Interest

The authors declare no conflicts of interest.

## Data Availability

The authors have nothing to report.
